# Risk factors of negative prostate biopsies: A CHAID decision tree analysis

**DOI:** 10.1002/bco2.70162

**Published:** 2026-02-02

**Authors:** Pierre Allainguillaume, Coralie Uthe‐Spencker, Laura Larnaudie, Mathilde Sibony, Anthony Kanbar, Albert Semaan, Nicolas Barry Delongchamps, Michael Peyromaure, Julien Anract

**Affiliations:** ^1^ Urology Department Cochin Hospital, APHP Paris France; ^2^ Pathology Department Cochin Hospital Paris France; ^3^ INSERM U1151, INEM, Université Paris Cité Paris France

**Keywords:** prostate biopsies, prostate cancer, risk factors

## Abstract

**Objectives:**

This study aimed to perform a risk analysis of any prostate cancer (Pca) and of clinically significant prostate cancer (csPCa) in a contemporary cohort of prostatic biopsies.

**Materials and Methods:**

We conducted a retrospective analysis of patients who underwent prostate biopsies in our centre between December 2020 and December 2022. We calculated Pca and csPCa rate (ISUP grade ≥2). Univariate and multivariate regression models were constructed to assess independent predictive factors for Pca and csPCa. We used *χ* ^2^ automatic interaction detection (CHAID) for decision tree analysis.

**Results:**

We included 255 patients in the analysis, of whom 69.8% had positive biopsies for Pca and 36.9% for csPCa. Multivariate analysis found PSA density (PSAd) (OR = 1.001) (1.000; 1.001), PIRADS score (OR = 1.393) (1.234; 1.571) as independent predictive factors of csPCa. For the detection of any PCa, CHAID analysis revealed that patients with PIRADS score ≤4 doubled the risk of negative biopsies (from 22.6% to 54.3%) when the prostate volume was >46 mL.

**Conclusion:**

For patients with a PIRADS ≤4, a large prostate volume (>46 mL) was a predictor of negative biopsies, independently of PSAd. MRI interpretation and targeting in these patients should therefore be performed with particular caution.

## 1 INTRODUCTION

Prostate cancer represents the second most common cancer in men worldwide, with an incidence of 1.4 million newly diagnosed cases in the year 2022.^1^ Currently, the prostate‐specific antigen (PSA) remains the primary screening and diagnostic test used in the suspicion of prostate cancer (PCa). However, it is not a cancer‐specific marker and can be elevated in several benign conditions.^2^, ^3^ Over the past decade, the detection of PCa has evolved towards clinically significant prostate cancer (csPCa) due to the spread of magnetic resonance imaging (MRI) and targeted biopsies of suspicious lesions.^4^ The PRECISION study^5^ and MRI First 01 study^6^ enabled to change the recommendation, highlighting improved detection of csPCa and reduced detection of nonsignificant prostate cancers (ncsPCa) (ISUP 1) in patients undergoing a series of targeted biopsies after MRI. The switch from systematic to fusion biopsies allowed drastic increased in detection rate of cancer in patient undergoing prostate biopsies. For the detection of any PCa, this switch has raised detection rates from 40 to 65%–70%.^7^, ^8^, ^9^, ^10^, ^11^ For csPCa, the detection rate increased to 45%.^7^ These progress have led to a decrease in the number of negative prostate biopsies, although the rate still remains around 30% to 35%.^7^, ^8^, ^9^, ^10^, ^11^

The first step in reducing the number of negative biopsies is to select patients who should undergo biopsies, i.e., those with a real risk of cancer. Hence, several studies assessed risk factors associated with PCa.^7^, ^12^, ^13^ Based on these works, the EAU Guidelines currently recommend performing prostatic biopsies for Prostate Imaging Reporting and Data System (PIRADS) 4 and PIRADS 5 target lesions.^14^ For PIRADS 3 lesions, prostate biopsy decision‐making is based on PSA density (PSAd), with cut‐off values of 0.15 to 0.2 ng/mL/cm^3^. Nevertheless, the real probability of detecting csPCa in patients with PIRADS 3 lesions, even when applying these thresholds, remains unclear.

Hence, the absence of PCa on histological analysis of biopsies is difficult to analyse in daily practice, due to the lack of data on the real cancer risk based on available tools such as PSAd and MRI findings. The management of patients with negative prostate biopsies directly depends on such risk assessment, in order to determine the appropriate timing to perform PSA control and/or MRI and/or repeat biopsies.

The aim of our study was to perform a risk analysis of any PCa and of csPCa in a contemporary cohort of prostate biopsies from a high‐volume centre, including an 18‐month follow‐up of patients with negative prostate biopsies to avoid the bias of missed target.

## 2 MATERIALS AND METHODS

### 2.1 Population

We included in our retrospective cohort patients who underwent systematic and targeted prostate biopsies between December 2020 and December 2022 in the Urology Department at Cochin Hospital in Paris. These patients either had a clinical suspicion of PCa (elevated PSA and/or abnormal digital rectal examination) or were under active surveillance for known prostate adenocarcinoma. All patients underwent prostate MRI prior to the biopsy procedure. Clinical, imaging, histological, treatment and follow‐up data were collected retrospectively. All patients included in the study were informed about the retrospective nature of the research conducted in the department and provided their consent. Patients with incomplete data were not included in the study. For each patient, the earliest biopsy performed during the inclusion period was considered as the T0 biopsy. All event times (including subsequent biopsies) were calculated from the date of this T0 biopsy. MRI data were based on the radiological report from the multiparametric MRI performed prior to the biopsy. Histological analysis was conducted by specialized uropathologists in the pathology department at Cochin Hospital. All prostate biopsies were performed by the same highly experienced operator (>1000 biopsy procedures). Biopsies were performed transrectally or transperineally using a system that included ultrasound and MRI image fusion software (Trinity®Koelis®). When a target lesion was identified on MRI, defined as PIRADS ≥3, patients underwent systematic biopsies combined with targeted biopsies. In the absence of a target, and when deemed appropriate by the referring surgeon, only systematic biopsies were performed. csPCa was defined as the presence of any tumoural gland with ISUP >1 in the biopsy series and ncsPCa was defined as ISUP 1 PCa in the biopsy series.

### 2.2 Statistics

Quantitative data were described using position parameters (median) as well as dispersion parameters (first quartile Q1 and third quartile Q3). Qualitative data were presented as number and percentage. Different proportions were compared using Fisher's exact test and means were compared using Student's *t*‐tests. Univariate and multivariable regression models were constructed to assess independent predictive factors for PCa and csPCa. Clinically and statistically significant variables were included in the multivariable model. A two‐sided p value <0.05 was considered statistically significant. The *χ* ^2^ automatic interaction detection (CHAID) algorithm was used, independently from the previous regression models, to perform decision tree analysis in which PCa or csPCa absence was the dependent variable. The decision tree was set to have a maximum of three levels and a minimum of 50 cases for each parent node, and any given split should not generate a child node with fewer than 50 cases; the significance level (a_merge_, a_split_ and *p*‐value) was set at 0.05. All statistical analyses were performed using SPSS statistical software (SPSS Inc., Chicago, IL, USA).

## 3 RESULTS

### 3.1 Demographic data

We included 255 patients in our study. The median age was 69 years (IQR 62–74) and the median prebiopsy total PSA was 8.00 ng/mL (IQR 4.35–15.05). The majority of patients underwent transrectal ultrasound‐guided biopsies. Patient and MRI characteristics are summarized in Table 1. In our cohort, 178 patients (69.8%) had positive biopsies for prostate adenocarcinoma, of whom 94 (52.8%) had ISUP grade ≥2 (Table 1). The median total PSA in the subgroup of patients with a positive biopsy for ISUP 1 was 6.71 (IQR 5.15–8.80) ng/mL, similar to the subgroup of patients with a negative biopsy (6.87 ng/mL, *p* = 0.11). In contrast, the median total PSA in the subgroup of patients with a positive biopsy for ISUP ≥2 was 10 (IQR 7.15–19.40) ng/mL.

**TABLE 1:** Patient, MRI characteristics and histological results for prostate biopsies (*n* = 255).

Parameter	Result
Age, year [IQR]	69 [62;74]
Total PSA, ng/mL [IQR]	8.00 [4.35;15.05]
Prostate volume, cm^3^ [IQR]	50 [35;70]
PSA density, ng/mL^2^ [IQR]	0.104 [0.021; 0.186]
History of prostate biopsies, *n* (%)	51 (20)
Approach, *n* (%)	
TR	190 (74.5)
TP	65 (25.5)
Target number, *n* (%)	
0 target	7 (3.7)
1 target	140 (73.7)
2 target	40 (21.1)
3 target	3 (1.6)
Target localisation, *n* (%)	
PZ	151 (85.3)
TZ	26 (14.7)
Target localisation, *n* (%)	
Base	48 (26.8)
Middle	81 (45.3)
Apex	50 (27.9)
Target size, mm [IQR]	11 [8;15]
PIRADS, *n* (%)	
3	49 (20.8)
4	132 (55.9)
5	55 (23.3)
Nb of systematic cores [IQR]	9 [8;10]
Nb of targeted cores [IQR]	3 [2;3]
Negative biopsies	77 (30.2)
Positive biopsies	178 (69.8)
ISUP 1	84 (47.2)
ISUP 2	50 (28.1)
ISUP 3	22 (12.4)
ISUP 4	13 (7.3)
ISUP 5	9 (5)
CsPCa	94 (36.9)

Abbreviations: csPCa, clinically significant prostate cancer; IQR, interquartile range; ISUP, international society of urological pathology; PIRADS, Prostate Imaging Reporting and Data System; PSA, prostate‐specific antigen; PZ, peripheral zone; TP, Transperineal; TR, Transrectal; TZ, transition zone.

### 3.2 Multivariable analysis

All factors identified as predictive of csPCa in univariate analysis were included in the multivariable analysis of predictive factors for csPCa (Table 2a). All the factors identified as predictive of any PCa in univariate analysis were included in the multivariable analysis of predictive factors for any PCa (Table 2b).

**TABLE 2a:** Multivariable analysis for factors associated with diagnosis of csPCa.

Variable	OR (95% CI)	*p*‐value
Age, year	0.997 (0.988; 1.005)	0.427
PSA, ng/mL	1.000 (0.999; 1.000)	0.236
PSA density	**1.001 (1.000; 1.001)**	**0.002**
Target number	1.088 (0.926; 1.279)	0.303
TP approach	**0.780 (0.619; 0.983)**	**0.036**
Prostate volume	1.000 (0.999; 1.001)	0.767
Target size	1.006 (0.996; 1.017)	0.242
PZ	**1.225 (1.006; 1.492)**	**0.044**
PIRADS	**1.393 (1.234; 1.571)**	**<0.001**

Abbreviations: CI, confidence interval; OR, odds ratio; PIRADS, Prostate Imaging Reporting and Data System; PSA, prostate‐specific antigen; PZ, peripheral zone; TP, transperineal.

**TABLE 2b:** Multivariable analysis for factors associated with diagnosis of any PCa.

Variable	OR (95% CI)	*p*‐value
Age, year	1.004 (0.996; 1.013)	0.320
PSA density	**1.000 (1.000; 1.001)**	**<0.001**
Target number	**1.188 (1.022; 1.354)**	**0.027**
TP approach	1.135 (0.896; 1.373)	0.266
Prostate volume	**0.999 (0.998; 1.000)**	**0.039**
Target size	1.000 (0.880; 1.011)	0.941
PZ	0.894 (0.691; 1.096)	0.300
PIRADS	**1.214 (1.090; 1.338)**	**<0.001**

Abbreviations: PSA, prostate‐specific antigen; TP, transperineal; PZ, peripheral zone; PIRADS, Prostate Imaging Reporting and Data System; CI, confidence interval; OR, odds ratio.

### 3.3 CHAID analysis

**FIGURE 1:**
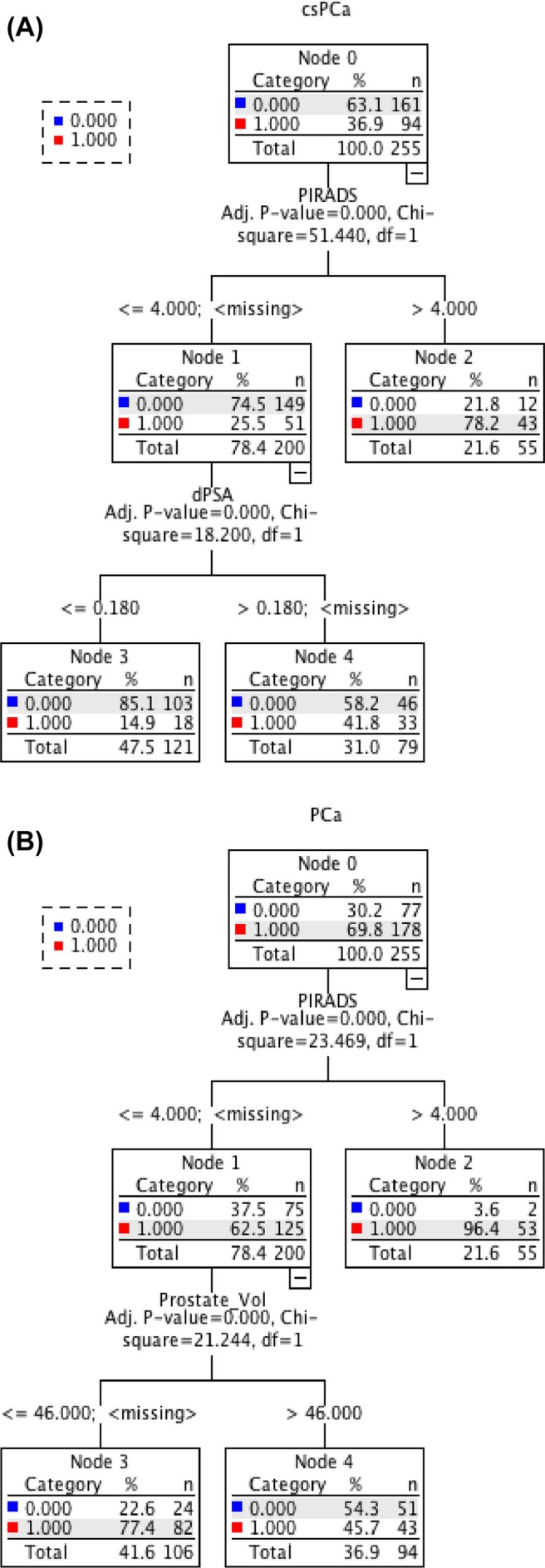
(a) Decision tree for csPCA probability according to the *χ* ^2^ automated interaction detection algorithm. CsPCa, clinically significant prostate cancer; PIRADS, prostate imaging reporting and data system; PSAd, prostate‐specific antigen density. (b) Decision tree for any PCa probability according to the *χ* ^2^ automated interaction detection algorithm. CsPCa, clinically significant prostate cancer; PIRADS, prostate imaging reporting and data system; PSAd, prostate‐specific antigen density.

We conducted CHAID decision tree analysis to identify the best cut‐off points for csPCa and for any PCa (Figure 1a,b). CsPCa and any PCa were included as the dependent variable in each tree, and all the factors included in the correspondant multivariable analysis were used as independent variable. The classification accuracy was 75.3% and 69.8% for csPCa and any PCa, respectively, and misclassification rate was 24.7% and 30.2% for csPCa and any PCa, respectively. The tree included three terminal nodes and two depth levels. For the detection of csPCa, PSAd and PIRADS score were decisive variables for classification, and two risk levels were created. For the detection of any PCa, PIRADS score and prostate volume were decisive variables for classification, and two risk levels were created. CsPCa accounted for 25.5% of the patients with a PIRADS score ≤4 (*χ* ^2^ = 51.4; *p* < 0.001). For these patients, the probability of csPCa decreased to 14.9% if they have a PSAd ≤0.18 ng/mL/cm^3^ (*χ* ^2^ = 18.2; *p* < 0.001). For patients with a PIRADS score of 5, the csPCa probability was 78.2% (*χ* ^2^ = 51.4; *p* < 0.001). And for patients who had a PIRADS score ≤4 and a PSAd >0.18 ng/mL/cm^3^, the csPCa probability was 41.8% (*χ* ^2^ = 18.2; *p* < 0.001).

## 4 DISCUSSION

The identification of risk factors for csPCa is crucial in the management of patients with an indication for prostate biopsy, to potentially repeat biopsies in patients with negative or nonsignificant prostate cancer and significant risk factors of csPCa. Prostate biopsies, while necessary to diagnose, remain invasive interventions. They are associated with potential adverse effects, notably infectious complications.^15^, ^16^, ^17^ Even in the absence of complications, biopsies can cause anxiety and discomfort for patients. In our cohort, multivariable analysis identified four independent predictive factors for the detection of csPCa: PSAd, biopsy approach (transperineal vs. transrectal), anatomical location of the MRI target in the peripheral zone and PIRADS score. These results are concordant with the literature.^7^, ^12^, ^13^ The CHAID analysis identified PSAd and PIRADS as the key factors to classify patients regarding their risk of csPCa. In this tree, PIRADS 5 was associated with a proportion of negative biopsies for csPCa around 20%. This is concordant with the literature, with major studies evaluating the global csPCa probability at 85% for PIRADS 5 lesions.^18^ For patients with PIRADS 3 or 4, the proportion of negative biopsies increased from 58.2% to 85% for patients with a PSAd <0.18 ng/mL/cm^3^. These findings are slightly different from the cut‐off of 0.15 ng/mL/cm^3^ proposed by EAU guidelines, reflecting that we are analysing a cohort of patients with an indication of biopsies, that is, with a median PSAd >0.15 ng/mL/cm^3^ in most cases. Hence, this PSAd cut‐off of 0.18 ng/mL/cm^3^ could be relevant to evaluate a posteriori the csPCa risk of patients with prostate biopsies negative for csPCa. These CHAID‐derived thresholds may assist clinicians in avoiding unnecessary biopsies in patients with PIRADS score ≤4 and PSAd ≤0.18 ng/mL^2^, who exhibit a low likelihood of clinically significant cancer detection. However, these decision rules warrant validation in prospective or multicentre cohorts before they can be implemented in routine practice.

Other studies identified PSAd as a predictive factor for csPCa in patients having prostate biopsies.^19^, ^20^, ^21^ A meta‐analysis showed that the csPCa risk in patients with PIRADS 3 lesions was 12% for a PSAd <0.15 ng/mL/cm^3^ and decreased to 6% for PSAd <0.1 ng/mL/cm^3^, but there was no information for PIRADS 4 and 5 lesions specifically.^21^ Mjaess et al. defined the PSAd cut‐off to 0.13 ng/mL/cm^3^ for PIRADS 3 lesions^19^ to avoid prostate biopsies. Pellegrino et al. defined the PSAd cut‐off to 0.10 ng/mL/cm^3^ in the same population and concluded that for patients with PIRADS 4 and 5 lesions on prebiopsy MRI, prostate biopsies were mandatory due to a CsPCa risk of more than 40%.^20^ Our results present a risk stratification based on a population with biopsies indication. Interestingly, the model proposed a PSA cut‐off for PIRADS 3 and 4 lesions in the same group.

Interestingly, other factors were identified as independent predictive factors of csPCa but were not statistically significant in the CHAID analysis. Notably, the TP approach was associated with a lower detection of csPCa in our cohort. Literature remains discordant about the potential higher detection rate of the TP approach.^15^, ^16^, ^22^ However, the small number of patients and the design of our work do not allow us to analyse these results. In addition, the location of the tumour in the peripheral zone was also associated with the detection of csPCa. This is in concordance with the literature, showing that around 70%–80% of tumours are located in the peripheral zone. Furthermore, the difficulty in analysing target lesions in the transition zone could induce a higher false‐positive MRI lesions in this particular area.

Our second analysis identified the following factors as independent predictive factors to detect any PCa: PSAd, target number, PIRADS and prostate volume. Interestingly, these factors were different from the analysis with csPCa as the dependent variable. Nonetheless, PSAd and PIRADS score were strongly associated with the detection of any PCa with the lowest *p*‐values (<0.001). Interestingly, the TP approach and the location in the peripheral zone were not predictive of any PCa but only of csPCa. In addition, the number of targets was predictive of the detection of any PCa but not associated with the detection of csPCa. Finally, prostate volume remained a significant variable in the multivariate analysis of the detection of any PCa, suggesting that prostate volume was significant independently from PSAd. Indeed, these results were confirmed with the CHAID analysis, which showed PIRADS and prostate volume as the more relevant factors to classify patients for the risk of detection of any PCa. In this tree, the risk of negative biopsies doubled (from 22.6% to 54.3%) when the prostate volume was >46 mL. We can suggest two hypotheses to explain these findings:

1. First, we can suppose that a high prostate volume increases the risk of missing the target, due to the technical difficulty of targeting in a great volume, or due to the potential error during the segmentation of a high‐volume prostate.
‐Second, we can suppose that a high prostate volume is mainly due to benign prostatic hyperplasia, inducing two phenomena: higher PSA increasing the biopsy indication, and development of transition zone, which is often heterogenous, leading to overestimation of the presence of a target, and hence false‐positive MRI.

Our decision tree model was constructed using the CHAID method, which offers interpretability and clarity by identifying key thresholds (e.g., PSAd = 0.18 ng/mL/cm^3^, prostate volume = 46 mL). This simplicity makes the model directly actionable in a clinical context. Nevertheless, CHAID may not fully capture more complex, non‐linear interactions among predictors, especially when compared to ensemble methods. Hence, to enhance the robustness and generalizability of our findings, external validation in independent cohorts should be conducted, employing more sophisticated algorithms, such as Random Forest.

In our cohort, the overall detection rate was 69.8% and 36.9% for CsPCa. In the literature, the overall detection rate is similar, ranging between 66% and 73%. Regarding the detection rate of CsPCa, the literature shows considerable variability between studies, with rates ranging from 33.5% to 72%.^7^, ^8^, ^9^, ^10^, ^11^ The variability in detection rates reported in the literature can be explained by the heterogeneity of included patients, biopsy approaches, operator expertise and the diversity of imaging and fusion systems used. The differences observed with our cohort can be attributed to the inclusion of patients already under active surveillance, unlike these studies. This may have increased the detection rate of nonsignificant cancers while reducing the proportion of significant cancers. Additionally, in Winoker et al. study, patients were older, and the proportion of patients with PIRADS 5 lesions was higher, which may partly explain the higher ISUP ≥2 detection rate compared to other studies.^11^

Our study included a follow‐up of negative biopsies to avoid the potential bias of missed targets, as it was our main subject of interest. During this 18‐month follow‐up, only three patients underwent second positive prostatic biopsies. Considering these three patients as initial positive biopsies (csPCa or ncsPCa) did not modify our results (multivariable and CHAID analysis). However, due to the retrospective design of the study, we can hypothesize that some patients did not undergo control biopsy due to stable PSA evolution and had actually a missed target, including a bias in our study. A longer follow‐up or the inclusion of these patients in a prospective protocol including systematic control biopsies according to the decision tree would answer that question. However, we believe that this retrospective design in a centre with high experience in prostatic biopsies offers results close to the reality.

Our study has limitations due mostly to its retrospective, single‐centred design and modest cohort size. The use of a single highly experienced operator to do all biopsies is another limitation in our study as it may limit its generalizability. The MRI data were based on radiology reports, which introduce potential variability in image interpretation and PIRADS scoring, and may therefore impact the resulting CHAID classification. However, all MRI examinations were performed in a high‐volume centre with standardized reporting practices, which likely helped to minimize such variability. We also chose to include patients with a history of endoscopic treatment for benign prostatic hyperplasia, which may induce bias in the interpretation of PSAd.

## 5 CONCLUSION

PIRADS and PSAd were independent predictive factors of negative biopsies for csPCa: For patients with a PIRADS score of 3 or 4, a PSAd ≤0.18 ng/mL/cm^3^, increased the proportion of negative biopsies from 58% to 85%. For the detection of any PCa, patients with PIRADS ≤4 lesions doubled the proportion of negative biopsies when prostate volume exceeded 46 mL. MRI interpretation and targeting in these patients should therefore be performed with particular caution.

## AUTHOR CONTRIBUTIONS

P.A. and J.A. conceived and designed the study. P.A. and J.A. drafted the manuscript. J.A. performed the statistical analyses. C.U.‐S., L.L., M.S., A.K., A.S., and N.B.D. contributed to data collection and data curation. M.P. and N.B.D. contributed to patient recruitment and provided critical clinical input. M.P. and J.A. supervised the study. All authors contributed to the interpretation of the data, critically revised the manuscript for important intellectual content, and approved the final version of the manuscript.

## CONFLICT OF INTEREST STATEMENT

The authors declare no conflicts of interest.
